# IMRT clinical implementation: Prostate and pelvic node irradiation using Helios and a 120‐leaf multileaf collimator

**DOI:** 10.1120/jacmp.v3i4.2551

**Published:** 2002-09-01

**Authors:** C. H. Clark, C. D. Mubata, C. A. Meehan, A. M. Bidmead, J. Staffurth, M. E. Humphreys, D. P. Dearnaley

**Affiliations:** ^1^ Joint Department of Physics The Royal Marsden NHS Trust Fulham Road London SW3 6JJ United Kingdom; ^2^ Academic Department of Clinical Oncology Institute of Cancer Research and The Royal Marsden NHS Trust Downs Road Sutton Surrey SM2 5PT United Kingdom; ^3^ Department of Radiotherapy The Royal Marsden NHS Trust Fulham Road London SW3 6JJ United Kingdom

**Keywords:** IMRT, radiotherapy, prostate, quality assurance

## Abstract

Dynamic intensity modulated radiation therapy (IMRT) to treat prostate and pelvic nodes using the Varian 120‐leaf Millennium multileaf collimator (MLC) has been implemented in our clinic. This paper describes the procedures that have been undertaken to achieve this, including some of the commissioning aspects of Helios, verification of the dynamic dose delivery, and quality assurance (QA) of the dose delivered to the patient. Commissioning of Helios included measurements of transmission through the 120‐leaf MLC, which were found to be 1.7% for 6 mV and 1.8% for 10 MV. The rounded leaf edge effect, known as the dosimetric separation, was also determined using two independent methods. Values of 1.05 and 1.65 mm were obtained for 6 and 10 MV beams. Five test patients were planned for prostate and pelvic node irradiation to 70 and 50 Gy, respectively. Dose and fluence verification were carried out on specially designed phantoms and dose points in the prostate were measured to be within 2.0% (mean 0.9%, s.d. 0.6%) of the calculated dose and in the nodes within 3.0% (mean 1.6%, s.d. 1.1%). Following the results of this commissioning and implementation study, we have started to treat men with a target volume including the prostate and pelvic nodes using Helios optimized dynamic IMRT delivery in a dose escalation protocol.

PACS number(s): 87.53.–j, 87.90.+y

## INTRODUCTION

The use of intensity modulated radiation therapy (IMRT) to improve dose conformity delivered using multileaf collimators (MLC) is increasing in radiation therapy clinics. The availability of optimization and inverse planning tools has facilitated the production of modulated beams and the delivery of these beams can be achieved using either static or dynamic leaf geometries.

There are different challenges to solve in the two delivery techniques. Although there is a school of thought that believes the static multisegment technique (“step and shoot”) to be easier to implement, there are arguments in favor of full leaf motion during beam‐on (dynamic MLC). In static techniques, the delivered fluence is divided into several intensity levels to reduce the segments to a manageable number. The appeal in the static techniques is that there is more apparent control during delivery in terms of leaf positioning and dose delivered at treatment as the output for each segment can be verified independently prior to treatment. With a comprehensive patient verification system, the individual segments may be delivered with confidence. The time taken to deliver these multiple static fields, however, depends on the between‐segment transition and verification time. Although the beam‐on‐time of the individual segments is small, the overhead time needed to download and verify the fields can lengthen the overall treatment times.

In dynamic techniques, verification of the output or monitor units is commonly carried out for whole field/plan delivery. This can be done for multiple dose point measurements using an ionization chamber (or diode chamber arrays) in conjunction with film measurements to obtain planar doses. The verification of the leaf positions with dose can be monitored using films, electronic portal imaging (EPI), or analysis of vendor provided MLC log files. The choice between using static and dynamic delivery depends on type and capabilities of planning and delivery hardware systems, as well as individual experience and confidence in the delivery system, i.e., the MLC and its monitoring system. Our experience with the Varian MLC and the experience from other centers (Memorial Sloane Kettering Cancer Center, New York, and Charite Hospital, Berlin) led us to decide to use dynamic delivery techniques for our first IMRT treatments. There are several excellent references discussing various individual aspects of IMRT which cover commissioning measurements,^1^ ^,^ ^2^ planning techniques,^3^ ^,^ ^4^ and treatment delivery quality assurance (QA).^5^ ^–^ ^8^ Our intention in this paper is to provide a description of the procedures we undertook, from the commissioning of a commercially available inverse planning system (CadPlan/Helios, Varian Medical Systems, Palo Alto, CA), to routine treatment of prostate and pelvic nodes. We have used the sliding window technique with the Varian 120‐leaf MLC.

The dosimetric characterization of the Varian MLC, required for dynamic delivery, has been described comprehensively in previous publications.^1^ ^,^ ^7^ However, a general overview of some of the parameters required for the initiation of IMRT treatments will be described in order to explain our choice of both the Helios and MLC parameters. The rationale behind the use of IMRT for pelvic nodes and prostate treatment has already been described in detail,^4^ and preliminary results from the RTOG trial 9413 suggest pelvic irradiation improves biochemical disease control compared with prostate radiotherapy alone.^9^

## MATERIALS

### A. Linear accelerator

The treatment delivery was carried out on a Varian 2100CD equipped with a 120 leaf MLC and an amorphous silicon (*a*‐Si:H) portal imaging system (PortalVision aS500, Varian Medical Systems) with photon beams of 6 MV. The MLC has 5 mm leaves covering a length of 20 cm in the middle and ten leaves of 10 mm on either side to complete the 40 cm length. Although a maximum speed of $3.0\;{\text{cms}}^{-1}$ can be achieved, a practical value of $2.5\;{\text{cms}}^{-1}$ is always used to conserve the motors. The maximum over‐travel across the midline of the leaves is 18.0 cm, but the maximum leaf span between the leading and trailing leaf edges is 14.5 cm. During an IMRT delivery, the MLC controller monitors and records the position of the leaves and the state of the beam in a log file every 50 ms. The system reads the current positions of all the leaves used in the field and records the square of the difference from the expected. The cumulative dose in Monitor Units (MU) and the beam state (whether the beam is on or “on‐hold”) are also recorded. Beam‐hold occurs when the data indicates that the leaves have not arrived at their intended positions for the given dose. The system then sends dummy pulses while waiting for the leaves to catch up.

### B. Computer planning system

Inverse planning was done on a CadPlan planning system using the Helios optimization module. CadPlan has been previously fully commissioned for 3D planning in our clinic. Therefore our task was to commission the Helios module to extend the CadPlan capabilities to IMRT. Helios uses the optimization algorithm of Spirou and Chui^10^ ^,^ ^11^ to produce “optimal” fluences for the different fields. The resolution of the optimum fluence is 0.25cm×leaf width (standard within Helios). The optimal fluences are converted to the “actual” fluences using the leaf motion calculator (LMC), which designs the leaf motion patterns. The LMC takes into account the various MLC parameters such as maximum leaf span, leaf speed, transmission, rounded end effects, and minimum leaf gaps. Since the *X* and *Y* jaws do not move during beam on, the maximum leaf span will determine how many carriage positions will be required to deliver the fluence for a given field width (*X* jaws). The field is split into multiple overlapping fields of the appropriate number of carriage or jaw positions. Although the leaf motions are not fully synchronized, the time of travel across the field is the same for all leaf pairs which helps to reduce the tongue‐and‐groove effect.^1^ The LMC produces the leaf motion files (.dva). The dose distribution for the actual fluences is calculated using the CadPlan single pencil beam (SPB).^12^

## METHODS AND RESULTS

### A. Configuration

Some of the initiation and configuration measurements required for both Helios and the linac have already been described in detail.^1^ ^,^ ^6^ The degree of accuracy of the calculated dose distribution with measurements depends on the configuration of the pencil beam algorithm and the MLC parameters entered into the planning system. The accuracy of the pencil beam algorithm kernels in modelling the scatter and field edges has an impact on the small field segments encountered in IMRT. The pencil beam kernels in the planning system are derived from measured profiles. Greater accuracy is therefore achieved by the use of detectors with high spatial resolution. A lack of resolution will result in under‐estimation of the contributions from very small segments. Furthermore, the regions of high dose peaks or low dose troughs present in the measured dose distributions might not be observed in the calculated fluences. A comparison of line profiles or planar dose maps between the calculated and measured data are always necessary to benchmark the accuracy of the calculation models. The accuracy of the algorithm in modeling edge effects affects the high dose gradient regions. Figures in Essers *et al.* ^1^ demonstrate such comparisons for different test geometries.

Configuration of the dynamic leaf motion parameters is crucial as this effects the calculated output and hence the dose delivered. In the conversion of the optimum to actual fluence, the LMC accounts for dose rate, minimum leaf gap, leaf transmission, and rounded leaf edge [“dosimetric leaf separation” (Varian term)]. The impact of these parameters on the dose delivery will be discussed briefly. The dose rate does not have an impact on the fluence, only the beam‐on time. However, increasing the dose rate increases the leaf speed and creates more frequent positional errors of greater magnitude. A dose rate of 600 MU/min was available on the linac; however, 400 MU/min was used for treatment delivery, as this is the machine default used for our conventional treatments. The leaf position tolerance was 2 mm.^6^ The minimum gap between the leaves minimizes collisions between the opposing leaves. This has an effect of raising the minimum dose in the field, but does not affect the delivered dose compared to the calculated. The other two parameters do affect the calculated dose compared to the delivered. The leaf transmission becomes crucial in IMRT fields because of the long beam‐on‐time with only MLC leaves covering part of the treatment field. Accurate values of leaf transmission are essential, as it can have an impact on the choice of other parameters such as the dosimetric leaf separation. A value for transmission that is too low will result in the calculated dose being too high compared with the measured dose. CadPlan requires only one value for the MLC transmission per energy. It has been shown^6^ that for 6 MV photons this value changes with depth and field size (range 1.8–2.4%). In our measurements the transmission through the leaves was found to be 1.6% and 1.8% at depths of 5 and 15 cm for 6 MV photons. The values were measured using an NACP chamber for a $12\times 12{\text{cm}}^{2}$ field with one set of MLC leaves positioned right across the field with the leaf ends joining under the opposite jaw. We took an average value of 1.7% for 6 MV photons. 10 MV photons showed less variation and measurements at 5 and 15 cm both gave values of 1.8%.

One observation to note when measuring the transmission though the leaves is that it is recommended to use an ion chamber with minimal low energy over response. The leakage/transmission through the leaves can be measured using film. However, obtaining the transmission values by taking the average of the scanned profiles is complicated by, for example, the normalization of open fields on different films. Also film has a well‐known over‐response at low energies. A more reliable method is to use a standard 0.6 cm^3^ ion chamber or a parallel plate chamber.^6^

**Figure 1:**
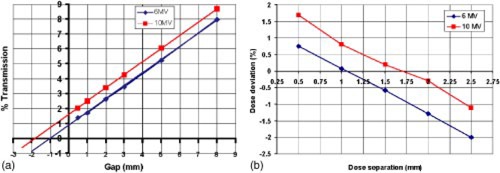
(Color) (a) The extrapolation of the graph of integrated dose versus leaf gap gives the offset of the leaf gap required as the dosimetric leaf separation value (6 and 10 MV were 0.97 and 1.79 mm, respectively). (b) The dose deviation (given as difference in measured and expected output) at a range of dosimetric leaf separations for 6 and 10 MV (6 and 10 MV were 1.05 and 1.65 mm, respectively).

The next important parameter to be determined is the leaf end effect or “dosimetric leaf separation.” This parameter effectively determines the positions of the leaves during dynamic delivery such that the increase in leakage between the rounded leaf ends is eliminated. There are two ways of deriving this parameter. One method is a variation of that proposed by LoSasso *et al.*,^6^ to verify the data by plotting net dose against gap width by moving slits of different leaf gaps and measuring the transmission. Dose is measured using a sliding window, which produces a uniform field, and an ionization chamber with a build‐up cap positioned off the end of the couch. The extrapolation of the graph of integrated dose versus leaf gap [shown in (Fig. 1a)] then gives the offset in leaf positioning required to eliminate the effect of the rounded leaf ends. Another way is to convert the optimum plan fluence to actual fluence using the LMC for a range of leaf gap separations. The dynamic leaf motion files from each of these plans is then used to deliver the same dose to an ionization chamber in a solid water phantom. A plot of the difference in measured and expected output from these plans is shown in (Fig. 1b) for 6 MV (blue) and 10 MV (red). If the value of dosimetric offset is too low, then the measured dose is greater than the expected dose (for example a value of 0.5 mm gives an increase in the measured dose of 0.75%). The required dosimetric leaf separation is taken to be the value giving zero dose deviation between measured and expected doses.

We found that values of dosimetric offset of 1.05 and 1.65 mm for 6 and 10 MV beams and transmission of 1.7% and 1.8% respectively gave no deviation between measured and CadPlan calculated doses. These values were verified by further tests of dose output at different points in a solid water phantom for a range of dose levels to various simple shaped volumes and field geometries.^1^ ^,^ ^2^

### B. Treatment planning

**Figure 2:**
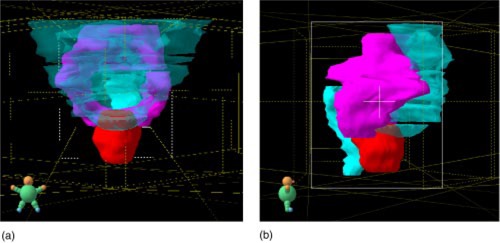
(Color) The volume to be treated and the OARs viewed from (a) the anterior and (b) the right lateral. The prostate PTV is shown in red, the pelvic lymph node PTV is shown in pink, and the bowel, bladder, and rectum OARs are shown in light blue.

The patients treated within this protocol have prostate cancer. They are considered at high risk of pelvic nodal involvement or have radiological or pathological evidence of nodal metastases. IMRT has been shown to reduce normal tissue irradiation without sacrificing target coverage compared to conventional techniques.^4^ ^,^ ^13^ Attempting to treat large volumes within the pelvis benefits from the sparing capabilities of IMRT.^4^ The small bowel is in close proximity to the pelvic nodes and the horse‐shoe shape of the nodes, as shown in Fig. 2, makes it a particularly difficult region to treat using conventional methods.

The treatment is designed to deliver a dose of 70 Gy to the prostate and 50 Gy to the seminal vesicles and pelvic nodes. In a phase 1 dose escalation trial the lymph node dose will rise to 55 and 60 Gy provided no significant acute or late toxicity is observed.

### C. Field setup

It has been shown^4^ that for prostate and pelvic node irradiation, reducing the number of beams from nine to five had no adverse effect on the planning target volume (PTV) coverage obtainable. It was also shown that the increase in volume of small bowel and colon irradiated to greater than 45 Gy was of the order of 2% when the number of fields was reduced from seven to five. It is unlikely that this small difference would be of clinical significance.

The benefit of reducing the number of fields is a reduction in the time taken for pre‐treatment quality assurance, dose and fluence verification and treatment delivery. The time required to carry out these processes is discussed later in this article.

**Figure 3:**
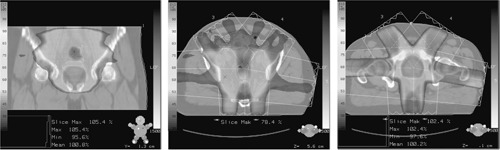
Typical examples of dose distribution in the prostate and pelvic nodes.

Gantry angles of 180° (posterior), 270° (right lateral), 325° (right anterior oblique), 35° (left anterior oblique), and 100° (left posterior oblique) have been chosen after evaluation of five different patients' treatment plans. The beams are spread out around the patient, provide good bowel sparing and are not opposing. Plans were optimized and calculated for both 6 and 10 MV photons; an example is shown in Fig. 3. No advantage was seen in using 10 MV and therefore we have chosen to use 6 MV in order to reduce the scattered radiation in the room.

**Table I:** The goal dose constraint protocol used for the prostate and pelvic node treatment. Targets are shown on the right, and organs at risk are shown on the left.

Structure	Dose (% ${\text{PTV}}_{1}$ dose)	Vol.	Structure	Dose	Vol.
Prostate ${\text{PTV}}_{1}$	≥63Gy(90%)	99%	Rectum^14^ ^,^ ^15^	≥45Gy	100%
	≥66.5Gy (95%)	95%		65 Gy	≤30%
	70 Gy (100%)	50%		70 Gy	≤15%
	73.5 Gy (105%)	≤5Gy		75 Gy	≤3%
Node and SV	≥45Gy(65%)	99%	Bladder^16^ ^,^ ^17^	50 Gy	≤50%
PTV_2_	≤47.5Gy (68%)	95%		60 Gy	≤25%
	50 Gy (71%)	50%		70 Gy	≤5%
Involved	≥50Gy(71%)	99%	Small bowel^18^	45 Gy	≤78cc
node	≤52.5Gy (75%)	95%		50 Gy	≤17cc
boost ${\text{PTV}}_{3}$	55 Gy (78%)	50%		55 Gy	≤14cc
				60 Gy	≤0.5cc
				65 Gy	≤0cc
			Femoral heads^19^	50 Gy	≤50%

### D. Optimization and dose constraints

The Helios inverse planning module requires that an optimal dose volume histogram (DVH) be designed for each target or organ at risk (OAR). The constraints applied to these DVHs consist of maximum and minimum doses and specific dose/volume points for each structure. Each of these points (including the maximum and minimum) then has a priority value of 0–100 assigned to it; where a high priority value means greater importance will be attached to achieving that particular dose constraint. Any of the dose or priority values can be adjusted during the optimization. Each structure requiring individual dose constraints must be contoured. The prostate CTV was considered to be the entire visible prostate and was grown to a PTV with a 1 cm margin. However, if the overlap between the PTV and rectum was large, then the posterior margin was reduced to 8 mm. The nodal CTV was expanded to a PTV with a uniform 5 mm margin. The overlap of PTV and OAR should be taken into consideration in the design of the DVH. For example, if the constraints to the anterior section of the rectum are to be different from those for the posterior part of the rectum, then these should be outlined as two separate structures.

The goal dose constraints we have used for the prostate and pelvic node treatment are given in Table I.^14^ ^–^ ^19^ These have been selected on the basis of a review of published data correlating dose‐volume constraints with late normal tissue complication. Optimization of the priorities used has been investigated to achieve acceptable PTV coverage while reducing the dose to the OARs. In general, the constraints for uniform prostate coverage are in competition with rectal sparing and the constraints for nodal coverage are in competition with bowel sparing. Experience of interaction with the dose constraints during optimization has increased our skill in producing improved DVH results.

It was found that the following order of interaction generally gives good results. The ideal DVH is designed with medium priorities on all the volumes. The optimization is allowed to run until an approximate solution is found. The priorities are then increased and the dose constraints tightened for the prostate PTV until acceptable coverage is achieved. Afterwards the priorities are increased on the rectum and the DVH points are moved to lower dose constraints to maximize the sparing while ensuring that PTV coverage is not lost. The priorities on the bowel are then increased to achieve acceptable bowel sparing and the DVH points on the lymph nodes moved to counteract the effects of the improved bowel sparing. In order to maintain 50 Gy at 50% for the lymph nodes, the 99% and 95% coverage may have to be sacrificed to achieve the bowel sparing. Any PTV/rectum overlap will limit the rectum sparing and any PTV/bowel overlap will limit the bowel sparing and the node coverage.

Helios then calculates a solution to these constraints and priorities and displays a DVH and an “ideal” fluence for each iteration of the calculation. Once the optimization has finished the user returns to the CadPlan workspace and the LMC converts the “ideal fluences” into “actual fluences.” These actual fluences are then used to calculate the dose distributions in CadPlan and an actual DVH can be calculated. Care must be taken to evaluate the dose distribution on individual transverse, sagittal, and coronal slices and to locate hot or cold spots, some of which may be acceptable depending on location.

Our experience has been that for the majority of patients the constraints in Table I are achievable. However, for patients with an overlap of nodal PTV and small bowel a compromise must be reached. In general this is reached by giving the bowel sparing greater importance than the node coverage (e.g., a patient with 2.3% overlap was given a bowel maximum of 45 Gy with 80% priority and the nodal minimum of 45 Gy with 70% priority). In such cases both options are planned and presented to a clinician.

For prostate and pelvic node treatment with five gantry angles, typical beam lengths are 16–18 cm and beam widths are 10–18 cm. Typical MUs are 95 (for a section of a split field) and 135 (for a maximum width single field). The prescribed dose is 200 cGy per fraction to the median of the prostate PTV

## QUALITY ASSURANCE OF DOSE AND INTENSITY MAPS A. Materials

**Figure 4:**
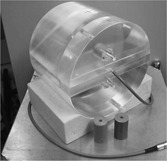
The phantom used with a pinpoint ionization chamber to make point dose measurements. Inhomogeneous/homogenous inserts can be interchanged.

A cylindrical phantom made of perspex has been designed for the purpose of IMRT dose verification, as shown in Fig. 4. It has insert positions for a pinpoint chamber [0.015cc pinpoint ionization chamber (PTW‐Freiburg, Freiburg, Germany)] used with a PTW‐Freiberg UNIDOS T10002 electrometer) and sections which can be replaced with inhomogeneous inserts.

A second verification phantom consists of 25cm×25cm slabs of solid water (GAMMEX RMI 457, Nottingham, UK) of varying thicknesses stacked together to form a cubic volume onto which the fluences are delivered and measured with film at different depths.

### B. Method

#### 1. Intensity map verification

For relative fluence verification each beam portal and its associated fluence are individually transferred in CadPlan onto a CT scan of the solid water slabs. The field is set to a gantry angle of 0° and the isocenter is set to a depth of 10 cm. The dose distribution is calculated in a coronal plane at isocenter. No renormalization is used so that the monitor units from the patient plan are the same in the fluence verification plan. This ensures that the dynamic delivery in terms of dose rate and leaf speed, calculated for the patient, is maintained.

**Table II:** Difference in % between measured and CadPlan doses from dose verification of five test patients' plans.

	Point 1 (superior prostate)	Point 2 (inferior prostate)	Point 3 (posterior prostate)	Point 4 (superior nodes)
Patient 1	−0.3	−0.1	−0.3	+2.8
Patient 2	+1.6	−1.7	−1.0	+2.2
Patient 3	−1.1	−1.5	−1.6	−0.3
Patient 4	+1.4	+0.5	+1.0	+0.6
Patient 5	+1.4	−0.2	+1.2	+2.0

**Figure 5:**
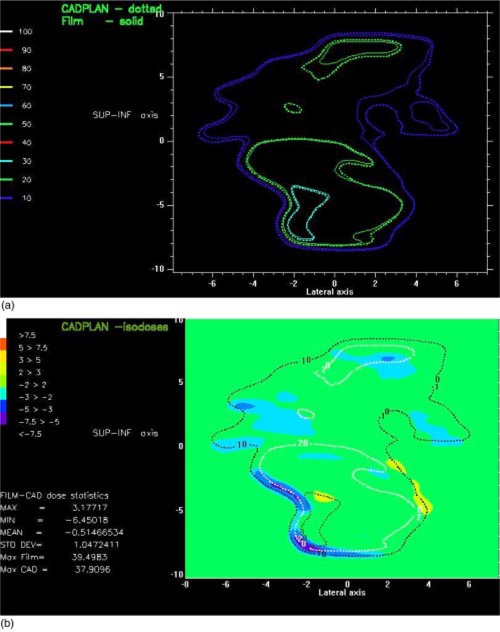
(Color) (a) Overlay of the isodoses, for fluence verification, from film and CadPlan at 10 cm depth. (b) Dose difference map of the isodoses from film and CadPlan at 10 cm depth.

Each field portal is delivered to a separate film (Kodak XV). The films are processed, scanned, and exported to our in‐house isodose comparison program with the 2D information binned in 2.5 mm dose pixels. The CadPlan isodoses calculated in a 2.5 mm dose matrix, at the same depth, are also exported to the isodose comparison program and the combined isodoses are displayed as overlays, as shown in (Fig. 5a), or dose difference maps, as shown in (Fig. 5b). In homogeneous regions the differences are within ±2% [shown in green in (Fig. 5b)]. Greater differences can be seen in regions of high dose gradient, which are accentuated by any difficulty in registering the two dose grids. The advantage of this simple comparison is that large differences can be visualized immediately. A more accurate method may be to combine dose and distance differences into a single value called the gamma index of Low *et al.* ^7^

#### 2. Dose (MU) verification

Currently MU verification is carried out by dose measurement. For absolute dose verification the entire patient plan is exported within CadPlan onto a CT scan of the cylindrical IMRT phantom. The plan is then calculated without renormalization, as it is for the fluence verification procedure. For each patient plan suitable measurement points need to be found in homogeneous dose regions with no dose gradients close by. Such suitable points are in the central region of the prostate PTV and in the superior region of the pelvic nodes. The CadPlan calculated doses at these points are noted and compared with the pinpoint chamber measured doses as a percentage difference.

### C. Results

Five test patients were outlined and planned. These plans were then transferred to the two QA phantoms for fluence and dose verification. Table II shows the results of the dose verification for the five test patients.

The points within the prostate were all measured to be within 2% of the calculated values and the points in the nodal region were all within 3%. The dose difference maps for the fluences were considered acceptable if the differences were less than 5%. Greater differences were accepted in the penumbra or in regions of steep dose gradient as difficulties in alignment of the two dose grids can cause large dose differences over small distances in these areas.

## PATIENT QUALITY ASSURANCE PRIOR TO TREATMENT

**Figure 6:**
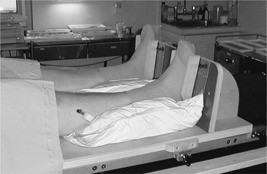
The immobilization footboard for the prostate and pelvic node patients.

It has been shown that leg immobilization increases pelvic setup reproducibility.^20^ A footboard was designed with this aim, as shown in Fig. 6, which fits onto the simulator, CT and treatment couches. This consists of a customized vacuum molded bag under the heels and ankles and a board to rest the feet against. The patients' feet can be repositioned in the same place with the aid of customizable location devices that fit on either side of the foot.

Patients were scanned with a comfortably full bladder, at 5 mm intervals using a spiral technique, using a GEC (General Electric) CT scanner. Before treatment commences the patient returns to the simulator (Ximatron, Varian Medical Systems) for an isocenter position check. The position is verified against printed digitally reconstructed radiographs (DRRs) produced from CadPlan for anterior and right lateral fields. Couch positions are acquired and entered into the VARiS record and verify system (version 6.1, Varian Medical Systems) as couch positions for the treatment fields. The footboard is relocated at a known position on the treatment couch each day to ensure fixed couch positions for each fraction. The films from the simulator are digitized using a Vidar scanner (VIDAR Systems Corporation, VA) and entered into VARiS as reference images for the isocenter check fields.

**Figure 7:**
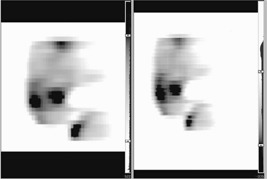
Comparison of actual Helios calculated fluence (left) and the portal image of the delivered fluence (right).

Actual fluence images are exported from CadPlan and attached as reference images to each of the treatment fields in vision (version 6.1, Varian Medical Systems). A “dummy run” of the treatment is carried out prior to treatment to verify gantry and couch positions. Images are also taken on the *a*: SiH portal imager during delivery of the fluences for each field and visually checked against the calculated fluences; see Fig. 7. No absolute difference is intended to be measured; the difference in the size and shape of the images exists because the reference image is calculated at isocenter (100 cm) and the portal image is measured at 140 cm. We perform this check to ensure that the fields are correctly delivered by VARiS. For each field the start position of the leaves as indicated by VARiS is also checked against the printout from CadPlan.

## PATIENT QUALITY ASSURANCE DURING TREATMENT

The highly conformal nature of this treatment requires that the setup uncertainty is the minimum achievable. This is especially true since there is also both inter‐ and intrafraction movement of the prostate within the body.^21^ The patients are asked to drink 2–3 glasses of water 30 min prior to scanning and treatment to ensure a comfortably full bladder. Anterior and lateral electronic portal images are taken over the first five days and then reviewed. If the average match error is greater than 3 mm, then the isocenter position is moved and the image retaken. However, if a systematic error is seen after three fractions the isocenter is adjusted immediately.

**Table III:** The time differences to carry out a prostate and pelvic node treatment delivered by IMRT compared with a two‐phase prostate treatment by conventional radiotherapy. R=radiographers, C=clinicians, and P=physicists.

Task	Time for IMRT treatment	Time for conventional treatment	Staff involved
Pre CT simulation, immobilization, and tattooing	15 min	15 min R
CT scanning	20 min	20 min	R
Outlining on hard copies	$\frac{1}{2}\;h$	$\frac{1}{2}\;h$	C
Outlining on CadPlan	$1\;\frac{1}{2}\;h$	$\frac{1}{2}\;h$	C
Planning	6 h	$1\;\frac{1}{2}\;h$ for Phase I	P
		1 h for Phase II
Checking plan	$\frac{1}{2}\;h$	$\frac{1}{2}\;h(\text{Ph I})+\frac{1}{2}\;h(\text{Ph II})$	P, C
Approving plan	$\frac{1}{4}\;h$	$\frac{1}{4}\;h(\text{Ph I})+\frac{1}{4}\;h(\text{Ph II})$	C
Isocenter check on simulator	$\frac{1}{2}\;h$	$\frac{1}{2}\;h$ (Ph I)	R
		$\frac{1}{2}\;h$ (Ph II)
QA	4 h	N/A	P
Checking VARiS	$\frac{1}{2}\;h$	$\frac{1}{4}\;h$	R
Dummy run of delivery	$\frac{1}{4}\;h$	N/A	R
Treatment delivery	$35\times \frac{1}{4}\;h(8\;\frac{3}{4}\;h)$	35×10min(5h50min)	R
Total	$23\;\frac{1}{2}\;h$	$12\;\frac{3}{4}\;h$	

## TIME AND RESOURCES

The time taken to plan, carry out the QA, and deliver the IMRT treatment has been monitored and recorded as shown in Table III. The times were compared with a standard three‐field prostate only treatment (we do not treat prostate with pelvic nodes in a conventional treatment). In total the extra time required for IMRT is 7.5 h for physicists (P),.50 h for clinicians (C) and 3 h for radiographers (R) per patient. Time is saved in the IMRT plan in checking (−.50h P and C), approving (−0.25h C), and in isocenter checks (−0.50h R) as the IMRT treatment is delivered in a single phase. The additional time for the IMRT plan is taken in the outlining (+1h C), planning (+3.5h P), and QA (+4h P).

As our experience and confidence grows we envisage the amount of QA required will be less and we also intend to implement new tools in the future with the aim of reducing the time taken to carry out the quality assurance. Currently the delivery time per treatment fraction is longer for the IMRT than for the conventional treatment (15 min compared with 10 min), however, we expect these to become comparable in the future as the technique becomes more routine. However, extra time is required for IMRT as our current protocols are three fields for conventional treatment and five fields for IMRT. We have found that extra personnel on the treatment unit were not required and the addition of IMRT treatment deliveries has not disrupted the normal working day.

## CONCLUSIONS

Following the results of this commissioning and implementation study treatment of prostate and pelvic nodes using Helios optimized dynamic IMRT delivery has started. We are currently working on implementing IMRT treatment for other sites and have found that our experience in the prostate and pelvic node treatment has accelerated the work required to do this.

## ACKNOWLEDGMENT

We work on the treatment machine for their enthusiasm in implementing this technique, Helen McNair for her advice, and Chris Fall for making the phantoms. This work was undertaken in the Royal Marsden NHS Trust, which received a proportion of its funding from the NHS Executive; the views expressed in this publication are those of the authors and not necessarily those of the NHS Executive. This work was supported by The Institute of Cancer Research, the Bob Champion Cancer Trust, the Cancer Research Campaign, and Varian Medical Systems.
